# Predictive model of length of stay in hospital among older patients

**DOI:** 10.1007/s40520-018-1033-7

**Published:** 2018-09-06

**Authors:** Radcliffe Lisk, Mahir Uddin, Anita Parbhoo, Keefai Yeong, David Fluck, Pankaj Sharma, Michael E. J. Lean, Thang S. Han

**Affiliations:** 1 Department of Orthogeriatrics, Ashford and St Peter’s NHS Foundation Trust, Guildford Road, Chertsey, Surrey, KT16 0PZ UK; 2 Institute of Cardiovascular Research, Royal Holloway, University of London, Egham, Surrey, TW20 0EX UK; 3 Department of Cardiology, Ashford and St Peter’s NHS Foundation Trust, Guildford Road, Chertsey, Surrey, KT16 0PZ UK; 4 School of Medicine, Dentistry and Nursing, New Lister Building, Glasgow Royal Infirmary, Alexandra Parade, Glasgow, G31 2ER UK; 5 Department of Endocrinology, Ashford and St Peter’s NHS Foundation Trust, Guildford Road, Chertsey, Surrey, KT16 0PZ UK

**Keywords:** Geriatrics, Discharge destination, Bed occupancy, Health economics, NHS, Frailty

## Abstract

**Background:**

Most National Health Service (NHS) hospital bed occupants are older patients because of their frequent admissions and prolonged length of stay (LOS). We evaluated demographic and clinical factors as predictors of LOS in a single NHS Trust and derived an equation to estimate LOS.

**Methods:**

Stepwise logistic and linear regressions were used to predict prolonged LOS (upper-quintile LOS > 17 days) and LOS respectively, from demographic factors and acute and pre-existing conditions.

**Results:**

Of 374 (men:women = 127:247) admitted patients (20% to orthogeriatric, 69% to general medical and 11% to surgical wards), median age of 85 years (IQR = 78–90), 77 had acute first hip fracture; 297 had previous hip fracture (median time since previous fracture = 2.4 years) and 21 (7.1%) had recurrent hip fracture, with median time since first fracture = 2.4 years. Median LOS was 6.5 days (IQR = 1.8–14.8), and 38 (10.2%) died after 4.8 days (IQR = 1.6–14.3). Prolonged LOS was associated with discharge to places other than usual residence: OR = 3.1 (95% CI 1.7–5.7), acute stroke: OR = 10.1 (3.7–26.7), acute first hip fractures: OR = 6.8 (3.1–14.8), recurrent hip fractures: OR = 9.5 (3.2–28.7), urinary tract infection/pneumonia: OR = 4.0 (2.1–8.0), other acute fractures: OR = 9.8 (3.0–32.3) and malignancy: OR = 15.0 (3.1–71.8). Predictive equation showed estimated LOS was 11.6 days for discharge to places other than usual residence, 15 days for pre-existing or acute stroke, 9–14 days for acute and recurrent hip fractures, infections, other acute fractures and malignancy; these factors together explained 32% of variability in LOS.

**Conclusions:**

A useful estimate of outcome and LOS can be made by constructing a predictive equation from information on hospital admission, to provide evidence-based guidance for resource requirements and discharge planning.

**Electronic supplementary material:**

The online version of this article (10.1007/s40520-018-1033-7) contains supplementary material, which is available to authorized users.

## Introduction

The population of older people in the UK is expanding. Among the 65.6 million population in 2016, there were 18% aged 65 years or over and 2.4% 85 years or over [1]. Ageing is accompanied with a number of chronic conditions that often require hospitalisations [2]. It is well recognised that hip fracture in older patients is a strong indicator of poor prognosis with annual mortality rates range between 15 and 30% [3–5]. Hip fracture in older patients is principally a marker of age-related frailty and co-existing morbidity, polypharmacy, cognitive decline and visual impairment, all of which contribute to falls and consequent fractures [6–9].

The majority of National Health Service (NHS) hospital bed occupants are older patients because of their frequent admissions and prolonged length of stay (LOS), exerting high burden on healthcare system. The National Audit Office reported that in 2014–2015, 62% of hospital bed days were occupied by older patients aged 65 years or older; this was an increase of 18% in emergency admissions since 2010–2011 [10]. About 2.7 million hospital bed days were found to be occupied by older patients who no longer needed acute treatment, equating to an excess of estimated gross annual cost to the NHS of approximately £820 million [10]. Prolonged LOS increases adverse health consequences to patients including sarcopenia [10] and increased risk of nosocomial infections [11–13]. These problems tend to perpetuate a vicious cycle of delay to discharge. It would be valuable for healthcare planning to identify both demographic and clinical factors that determine prolonged LOS, and to develop a simple evidence-based predictive equation to help manage discharge planning.

In the present study, we evaluated demographic and clinical factors as predictors of LOS in older patients in medical wards of a single NHS Trust and derived an equation to estimate LOS from these factors.

## Methods

### Study design, patients and setting

**Fig. 1:**
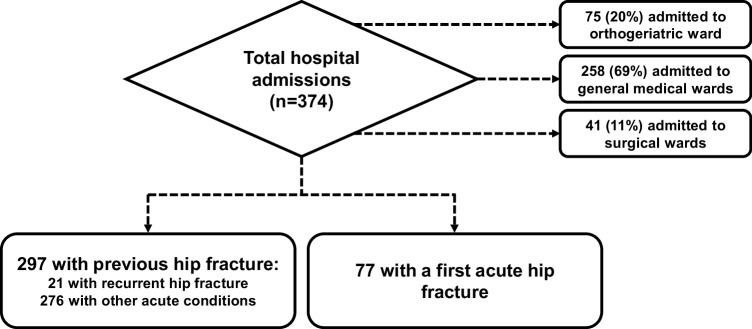
Flowchart of patient cohort investigated in this study

We conducted this study of a total of 374 older patients admitted to a single NHS Trust (Ashford and St Peter’s Hospitals NHS Foundation Trust) which serves a population of over 410,000 people living in the County of Surrey, UK. Figure 1 shows the flowchart of patients investigated in this study, 75 (20%) were admitted to the orthogeriatric ward, 258 (69%) to general internal medical wards and 41 (11%) to surgical wards between November 2014 and May 2016 (18 month period); 297 of these patients had a previous hip fracture (median time since previous fracture = 2.4 years). Among the patients with histories of previous hip fractures, 21 (7.1%) patients presented with a recurrent hip fracture and the remaining 276 patients presented with other acute conditions (Supplementary material). For comparison of outcomes between patients with a first fracture and those with a recurrent fracture, we included 77 patients with an acute first hip fracture within this 18-month study period who represent 15% of all patients admitted with acute hip fractures to our Trust (388 hip fractures per annum). We specifically selected this high-risk cohort of patients on the basis of their association with a wide range of co-existing conditions that have the greatest impact on the LOS in hospital, thus allowing the inclusion of the maximum number of explanatory variables for analyses.

The data on hip fracture were collected prospectively by a Trauma Coordinator for every patient admitted with a hip fracture as part of the National Hip Fracture Database [14] from the time of admission to discharge, comprising clinical characteristics and care quality as well as LOS during admission and discharge destination. Information on primary diagnosis at admission and pre-existing co-morbidities was identified from electronic record database by the disease codes classified by the International Classification of Diseases, Tenth Revision (ICD-10) [15]. To increase power of detection, we created the composite variable ‘acute infections’ comprising patients with pneumonia or urinary tract infection (UTI). Mental status at admission, assessed by abbreviated mental test score (AMTS) was also included in the analyses.

### Categorisation of variables

Dichotomisation was applied for all conditions according to their presence or absence. Prolonged LOS was derived from our data as patients who stayed over the period of highest fifth quintile of LOS: > 17 days in hospital. Destination on discharge was defined as either “discharged back to usual residence” or “discharged to places other than usual residence” including nursing homes, rehabilitation centres and residential homes. Impaired cognitive function was considered when AMTS ≤ 8 [16].

### Statistical analysis

Stepwise logistic regression was conducted to determine the significant predictors of prolonged LOS in hospital (dependent variable) from demographic factors including sex and destination on discharge and acute and pre-existing clinical conditions presented at admission (independent variables). Two logistic regression models were conducted, the first model was unadjusted (univariate) and the second adjusted (multivariate) for sex, and pre-existing major co-morbidities including ischaemic heart disease (IHD), atrial fibrillation, stroke, Parkinson’s disease, chronic obstructive pulmonary disease (COPD), malignancies, inflammatory bowel disease, diabetes mellitus and hypertension. Multiple linear regression was used to derive an equation to predict LOS in hospital from the aforementioned demographic and clinical factors. Because of this narrow age band in this cohort, age was not a significant contributing factor in the prediction model for LOS. Analyses were performed using SPSS V.22.0 (SPSS Inc, Chicago, IL, USA). The null hypothesis was rejected when *p* < 0.05.

## Results

Of 374 patients with median age of 85 years (IQR = 78–90), there were 127 (34%) men with median age of 84 years (IQR = 77–88) and 247 (66%) women with median age of 84 years (IQR = 75–88) who presented with 23 conditions and 8 pre-existing chronic conditions with 100 (26.7%) having 3 or more of these pre-existing conditions. 77 (20.6%) had acute first hip fracture and 297 (79.4%) had previous hip fracture; among those with previous hip fracture, 21 (7.1%) had recurrent hip fracture (Supplementary material). Median time since the previous to recurrent fracture was 2.4 years. During admission, 38 (10.2%) patients died after median LOS of 4.8 days. Among those who lived to be discharged, (*n* = 336) there were 115 (34.2%) men with median (IQR) age of 83 years (75–88) and 221 (65.8%) women with median age of 85 years (78.5–90). Median LOS was 6.5 years (1.8–14.8). There were 254 (75.6%) patients discharged back to usual residence and the remaining 82 (24.4%) to other places including nursing homes, rehabilitation centres and residential homes.

The adjusted risk of prolonged LOS was increased by threefold in those who were discharged to places other than usual residence, 10-fold in those admitted with acute stroke, sevenfold with first hip fracture and 10-fold with recurrent hip fractures, fourfold with acute infections, 10-fold with other acute fractures and 15-fold with malignancies (Table 1). Acute coronary syndrome (ACS) or COPD, diabetes, inflammatory bowel disease and or falls did not predict prolonged LOS.

**Table 1:** Prediction of prolonged LOS in hospital (upper quintile > 17 days) by discharge destination and primary diagnoses at admission is shown in the Supplementary Material using stepwise regression in all 374 patients

	Event rates and proportion (%) of patients with prolonged LOS				Logistic regression to predict the risk of prolonged LOS					
					Model 1: unadjusted^a^			Model 2: adjusted^b^		
	Event rates	%	*χ* ^2^	*p*	OR	95% CI	*p*	OR	95% CI	*p*
Discharge to usual residence (referent)	40/292	13.7	33.5	< 0.001	1	–	–	1	–	–
Discharge to other destinations	35/82	42.7			6.69	2.71–8.13	< 0.001	3.14	1.72–5.73	< 0.001
No acute stroke (referent)	63/348	18.1	11.9	0.001	1	–	–	1	–	–
Acute stroke	12/26	46.2			3.88	1.718.71	0.001	10.09	3.68–27.68	< 0.001
No acute first hip fractures (referent)	50/297	16.8	9.3	0.003	1	–	–	1	–	–
Acute first hip fractures	25/77	32.5			2.38	1.35–4.18	0.003	6.81	3.14–14.78	< 0.001
No recurrent hip fractures (referent)	67/353	19.0	4.5	0.039	1	–	–	1	–	–
Recurrent hip fractures	8/21	38.1			2.63	1.05–6.59	0.040	9.51	3.15–28.69	< 0.001
No acute infections (referent)	39/244	16.0	7.3	0.007	1	–	–	1	–	–
Acute infections (pneumonia or UTI)	36/130	27.7			2.01	1.20–3.37	0.008	4.00	2.10–7.95	< 0.001
No other acute fractures (referent)	68/355	19.2	3.5	0.061	1	–	–	1	–	–
Other acute fractures	7/19	36.8			2.46	0.93–6.49	0.068	9.82	2.99–32.30	< 0.001
No malignancy (referent)	71/365	19.5	3.4	0.084	1	–	–	1	–	–
Malignancy	4/9	44.4			3.31	0.87–12.65	0.080	15.01	3.14–71.77	0.001

Significant variables selected by regression are presented

^a^Model 1: unadjusted univariate logistic regression

^b^Model 2: adjusted multivariate stepwise logistic regression with adjustment for sex, and pre-existing co-morbidities including IHD, atrial fibrillation, stroke, COPD, malignancies, inflammatory bowel disease, diabetes mellitus and hypertension shown in the Supplementary Material

**Fig. 2:**
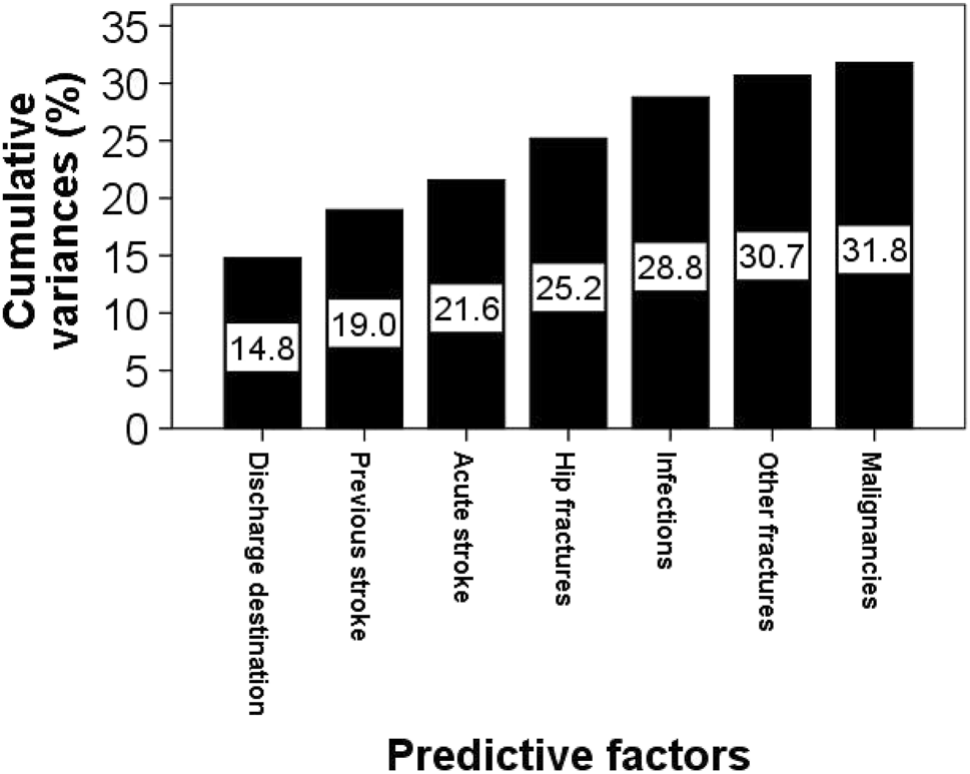
Cumulative variances in LOS in hospital explained by discharge destination and a number of clinical factors

A predictive equation was constructed showing estimated LOS to be 11.6 days for discharge to places other than usual residence, 15 days for acute or pre-existing stroke, 9–14 days for acute and recurrent hip fractures, acute infections, other acute fractures and malignancy. Estimated LOS for each of these factors were calculated and presented in Table 2 for future reference. These factors together explained 32% of total variances in LOS in hospital. The most powerful explanatory factor was discharge to places other than usual residence (15% of the variance), followed by stroke, recurrent hip fracture and infections, each of which explained between 2 and 4% of the variance (Fig. 2).

**Table 2:** Equation derived from multiple regression to predict LOS in hospital in 374 patients: LOS (days) = (9.1 × discharged to places other than usual residence) + (12.0 × pre-existing stroke) + (12.6 × acute stroke) + (6.6 × acute infections) + (7.6 × acute first hip fracture) + (11.8 × recurrent hip fracture) + (9.2 × other acute fractures) + (9.7 × acute malignancy) + 2.5 [total explained variance in LOS = 32.2%]

	Estimated LOS derived from multiple regression equation					Median (IQR) LOS (days)^c^
	Regression coefficients (*β*)	95% CI	Explained variance (%)	*p*	Estimated LOS (days)^a^	
Discharged to places other than usual residence^b^	9.1	6.2–12.1	14.6	< 0.001	11.6	14.9 (9.9–25.6)
Pre-existing stroke	12.0	5.3–18.6	4.2	< 0.001	14.5	15.8 (4.9–52.3)
Acute stroke	12.6	7.8–17.3	2.6	< 0.001	15.1	14.4 (4.0–34.8)
Acute infections	6.6	4.0–9.1	2.4	< 0.001	9.1	9.3 (4.1–17.1)
Acute first hip fractures	7.6	4.5–10.7	2.3	< 0.001	10.1	12.5 (7.3–19.5)
Recurrent hip fractures	11.8	6.6–16.9	2.9	< 0.001	14.3	13.6 (9.2–12.6)
Other acute fractures	9.2	4.0–15.8	1.9	0.001	11.7	10.5 (0.9–21.4)
Acute malignancies	9.7	3.7–14.6	1.1	0.014	12.2	15.0 (2.3–27.9)
(Constant)	2.5	0.7–4.4	–	0.008	–	–

The estimated LOS for a particular condition (1 in the presence and 0 in the absence of the condition) are presented in this column^a^ which is calculated using the general regression formula: estimated LOS (days) = 1 ×  *β* + 2.5, e.g., LOS for stroke = 1 × 12.6 + 2.5 = 15.1 days

^b^A further 9.1 days is added to LOS if the patient is discharged to places other than usual residence

^c^Median LOS, calculated from unadjusted data, are presented herein for comparison with estimated LOS

The adjusted risk of mortality was increased in patients who were admitted with ACS by sevenfold, with infection by 2.3-fold, with pre-existing cardiac arrhythmias by 2.4-fold and with pre-existing COPD by 2.2-fold (Table 3).

**Table 3:** Factors associated with increased risk of mortality

	Proportion (%) of mortality			Logistic regression to predict the risk of mortality					
				Model 1: unadjusted^a^			Model 2: adjusted^b^		
	%	*χ* ^2^	*p*	OR	95% CI	*p*	OR	95% CI	*p*
No acute coronary syndrome (referent)	17.1	5.1	0.026	1	–	–	1	–	–
Acute coronary syndrome	34.4			4.03	1.00–16.28	0.051	7.32	1.64–32.68	0.009
No acute infections (referent)	7.4	6.0	0.013	1	–	–	1	–	–
Acute infections (pneumonia or UTI)	15.4			2.28	1.16–4.49	0.017	2.26	1.11–4.58	0.025
No pre-existing atrial fibrillation (referent)	8.0	6.2	0.014	1	–	–	1	–	–
Pre-existing atrial fibrillation	17.2			2.39	1.19–4.82	0.015	2.41	1.17–4.97	0.017
No pre-existing COPD (referent)	7.8	4.2	0.033	1	–	–	1	–	–
Pre-existing COPD	14.5			2.00	1.02–3.3	0.044	2.22	1.08–4.57	0.030

^a^Model 1: unadjusted univariate logistic regression

^b^Model 2: adjusted multivariate stepwise logistic regression with adjustment for sex, and pre-existing co-morbidities including IHD, atrial fibrillation, stroke, Parkinson’s disease, COPD, malignancies, inflammatory bowel disease, diabetes mellitus and hypertension shown in Supplementary Material

Compared with patients with AMTS > 8, significantly greater proportions of those with AMTS ≤ 8 (impaired cognitive function) had stroke (4.5% vs. 11.6%, *χ*^2^ = 6.7, *p* = 0.010), infections (31.1% vs. 41.1%, *χ*^2^ = 3.5, *p* = 0.041), prolonged LOS (17.1% vs. 25.6%, *χ*^2^ = 3.8, *p* = 0.037, median LOS: 5.9 days vs. 9.3 days), and discharge to places other than usual residence (16.3% vs. 32.6%, *χ*^2^ = 13.0, *p* < 0.001).

## Discussion

In this comprehensive analysis of a relatively large cohort of the oldest group of patients in hospital, we have identified the most important determining factors of prolonged LOS. Our estimates of LOS from these factors provide multidisciplinary team crucial evidence-based guidance for discharge planning of older patients.

Current discharge planning among NHS hospitals follows published guidelines. The National Institute for Health and Clinical Excellence [17] recommends an integrated network of hospital-based and community-based multidisciplinary teams to provide coordinated support for older patients, from hospital admission through to discharge. Although there is national guidance and legislation relating to discharge practice and processes from acute hospitals, the local situation varies considerably. In the location of this study, there is an active multi-agency discharge group considering patients requiring complex discharges which involve post-hospital health or social care support. This group incorporates the acute provider, the community health provider, social services and continuing health commissioners. Although this collaborative group has improved cross-agency working there are still persistent delays in discharge of patients with complex needs. This has been mitigated to some extent in the orthogeriatric cohort by the introduction of a supported discharge team. To overcome these challenges faced by the NHS, the National Audit Office [10] has outlined further recommendations to better coordinate the central assurance and support for patient flow and discharge. These include collaboration between the Department of Health, NHS England and NHS Improvement to set out how discharge delays could be prevented and to minimise avoidable admissions and inappropriate prolonged LOS.

Our findings of the association between those who were discharged to places other than their usual residence are consistent with previous studies [18, 19]. The National Audit Office [10] estimated that about 1.15 million bed days were lost due to delayed transfers of care in acute hospitals during 2015 (up 31% since 2013) and found that 54% hospitals in their survey reported that discharge planning did not start soon enough to minimise delays for the majority of older patients. There are a number of factors that explain this delay such as high number of moves between wards [18]. Discharge delay has been shown to be influenced by socioeconomic and policy factors as well as decision-making process between interdisciplinary team members and patients [20]. A study by Gaughan et al. [21] has indicated that fewer delayed discharges were associated with hospitals with Foundation Trust status (semi-autonomous organisational units within the NHS in England which have a degree of independence from the Department of Health to decide locally how to meet their obligations) and a greater local provision of long-term care home beds. Analysis of clinical data such as those collected by clinical audits or registry-based clinical trials is an integral part of the delivery of care quality to identify and address factors associated with readmissions [22] and prolonged LOS [23]. However, prospective studies are required to quantify what it would take to increase the number of patients who returned to their usual place of residence and to assess whether that intervention would reduce their readmission rates and LOS.

Our findings are consistent with those from previous studies on the association between prolonged LOS with stroke [24, 25], UTI [26] and pneumonia [27], although some studies have found variable relationships between pneumonia and LOS [28]. The lack of significant association with some of the major conditions such as diabetes is perhaps surprising. This may be explained by the selection bias in this group of older individuals whereby those with the most severe conditions may have died prematurely before admission. There is increasing evidence that type 2 diabetes has relatively little clinical impact in the very older patients [29]. In our study, the increased rates of mortality among patients who were admitted with ACS and pneumonia and pre-existing arrhythmias and pre-existing COPD are likely to diminish the association between these conditions and LOS.

The rates of recurrent hip fractures (7.1%) over 2.4 years in the present study is similar to previously reported figures [5, 30]. Findings from our study of the increased risk of prolonged LOS among patients with recurrent hip fractures suggest a poor prognosis in these patients. Berry et al. [5] observed that 1-year mortality following an initial hip fracture was 15.9% which rose to 24.1% following a second hip fracture. Although the underlying explanation for this relationship is uncertain, it is probably due to a number of factors including the patients’ underlying frailty that requires longer time for recuperation but prolonged stay in hospital is associated with, and possibly leads to, more adverse consequences. It has been reported that a loss of about 5% of muscle strength per day of treatment in a hospital bed [10] and it is well recognised that older patients are particularly at increased risk of a number of nosocomial infections [12, 13]. Saviteer et al. studied over 4000 nosocomial infections in 2567 patients admitted to an acute-care hospital and estimated the daily infection rates in patients over 60 years to be 0.59% [11].

Patients with dementia have been shown to have longer LOS than those without dementia [31]. Although we did not have information on dementia as a diagnosis, the commonly used AMTS was available, which has been shown to be a good indicator of impaired cognitive function [15]. We observed that AMTS ≤ 8 was significantly associated with prolonged LOS and with discharge to places other than usual residence in unadjusted statistical model (Chi-squared test or univariate regression) while this association disappeared on adjustments with other clinical factors in multivariate regression model; these findings suggest a complex relationship whereby impaired cognitive function is a manifestation of other acute conditions such as stroke and infections, both of which were shown to be associated with low AMTS in the present study.

The strengths of the present study lie in its wide range of acute conditions which were identified by ICD-10 and adjusted for pre-existing co-morbidities. We focused only on a specific category of the oldest group of very specific and high-risk patient in hospital: our findings, therefore, should not be extrapolated uncritically to other populations. We suggest further studies are needed to examine all admissions including patients with no previous hip fractures as control group to eliminate the impact of previous hip fractures on other predictive factors. The emphasis of our paper was to provide a ‘worked example’ for a very high-risk patient group of a method to predict LOS. Different hospitals with different patient demographics will need to compute their own equation, modelling on the method we have demonstrated.

In conclusion, we have shown that it is possible to identify a number of factors which independently predict outcomes and LOS among this group of patients. Constructing a prediction equation using the approach we have described, based on information available on admission, provides a useful estimate of LOS. This is a simple way to identify the most important factors and to improve resource allocation and discharge planning.

## Electronic supplementary material

Below is the link to the electronic supplementary material.

Supplementary material 1 (DOCX 28 KB)
