# Supplementary material for: Predictive model of length of stay in hospital among older patients
Source: Aging Clin Exp Res. 2018 Sep 6;31(7):993–9. doi: 10.1007/s40520-018-1033-7 (PMC6589144; doi:10.1007/s40520-018-1033-7)
Supplement: Supplementary file 1 — Supplementary material 1 (DOCX 28 KB) [file 40520_2018_1033_MOESM1_ESM.docx]

**Supplementary Material.** Proportions of 374 patients presented with primary acute conditions at admission and their pre-existing comorbidities.

| **Primary acute conditions at admission** | **International Classification of Disease Tenth Revision (ICD-10)** | Number and proportion (%) of events |
| --- | --- | --- |
| Acute first hip fractures | S7200, S7240, S7220 | 77 (20.6%) |
| Acute recurrent hip fractures | S7200, S7240, S7220 | 21 (5.6)^†^ |
| Other fractures | I591, M8413, M966, N179, S524 | 19 (5.1%) |
| UTI | N390 | 85 (22.7) |
| Pneumonia | J22X, J180, J189 | 52 (13.9%) |
| UTI or pneumonia | Composite variable created for analysis | 130 (34.8%) |
| Sepsis | A415, A419 | 4 (1.1%) |
| Cellulitis | L031 | 3 (0.8%) |
| Stroke | G459, I619, I635, I638, I639, I678, I693, I951 | 26 (7.0%) |
| COPD | J440, J441, J448, J449 | 16 (4.3% |
| Inflammatory bowel disease | K509, K515, K519 | 1 (0.3%) |
| Heart failure | I500, I501, I509 | 7 (1.9%) |
| Acute coronary syndrome (unstable angina or myocardial infarct) | I200, I219, I213, I252 | 10 (2.7%) |
| Angina pectoris | I209 | 2 (0.5%) |
| Acute ischaemic heart disease/atherosclerotic heart disease | I249, I251 | 4 (1.1%) |
| Atrial fibrillation | I48X, I480, I489 | 6 (1.6%) |
| Malignancies | C61X, C165, C169, C509, C674, C719, C180, C184 | 9 (2.4%) |
| Diabetes mellitus | E109, E115, E116, E119 | 4 (1.1%) |
| Falls | R296 | 5 (1.3%) |
| Orthostatic hypotension | I951 | 4 (1.1%) |
| Parkinson’s disease | G20X | 3 (0.8%) |
| Acute kidney injury | N179 | 4 (1.1%) |
| Anaemia | D649 | 3 (0.8%) |
| Hypertension | I10X | 1 (0.3%) |
| Impaired cognitive function (AMTS ≤8) | Based on Abbreviated Mental Test Score | 129 (34.5) |
|  |  |  |
| **Pre-existing conditions** |  |  |
| Previous hip fractures | S7200, S7240, S7220 | 297 (79.4%) |
| Ischaemic heart disease | I249 | 102 (27.3%) |
| Atrial fibrillation | I48X, I480, I489 | 87 (23.3%) |
| Stroke | G459, I619, I635, I638, I639, I678, I693, I951 | 12 (3.2%) |
| COPD | J440, J441, J448, J449 | 131 (35.0%) |
| Inflammatory bowel disease | K509, K515, K519 | 6 (1.6%) |
| Diabetes mellitus | E109, E115, E116, E119 | 52 (13.9%) |
| Hypertension | I10X | 196 (52.4%) |
| Malignancy | C61X, C165, C169, C509, C674, C719, C180, C184 | 2 (0.5%) |
| **Three or more pre-existing conditions** | **--** | **100 (26.7%)** |

^†^Proportion of recurrent hip fractures (21 cases) relative to previous hip fractures (297 cases) = 7.1%.
